# Supplementary material for: Newborn MTHFR rs1801133 Variant and Extremely Low Birth Weight: A Case–Control Study and Meta-Analysis
Source: Genes (Basel). 2025 Oct 13;16(10):1192. doi: 10.3390/genes16101192 (PMC12562856; doi:10.3390/genes16101192)
Supplement: Supplementary file 1 [file genes-16-01192-s001.zip › genes-3858157-supplementary.pdf]

*Supplementary Materials*

# Newborn *MTHFR* rs1801133 Variant and Extremely Low Birth Weight: A Case–Control Study and Meta-Analysis

**Bartosz Skulimowski [1], Anna Durska [2], Alicja Sobaniec [3], Anna Gotz-Więckowska [1] and Ewa Strauss [2,*]**

[1] Department of Ophthalmology, Poznan University of Medical Sciences, 60-569 Poznan, Poland; skulimowskibartosz@gmail.com (B.S.); agotzwieckowska@ump.edu.pl (A.G.-W.)

[2] Institute of Human Genetics of the Polish Academy of Sciences, 60-479 Poznan, Poland; anna.durska@igcz.poznan.pl

[3] Department of Neonatology, Gynecology and Obstetrics Clinical Hospital, Poznan University of Medical Sciences, 60-535 Poznan, Poland; alicja.sobaniec@gmail.com

* Correspondence: strauss@man.poznan.pl

**Supplemental Table S1.** Comparison of clinical parameters between Study Cohort A and B.

| Parameter | | Study Cohort A (2009 - 2014) n=164 | Study Cohort B (2015 - 2020) n=213 | B *vs.* A p-value or OR (95% CI), p-value |
|---|---|---|---|---|
| Gestational age [weeks] | Mean (SD) | 27.8 (1.8) | 27.8 (2.3) | 0.936 |
| | Range | 24 - 31.9 | 22-31.9 | |
| | <28 weeks), n (%) | 68 (41.5) | 84 (39.4) | 0.691 |
| Birth weight [g] | Mean (SD) | 1,145.7 (301.6) | 1,067.3 (332.8) | 0.018 |
| | Range | 490 – 1,960 | 432 – 2,340 | |
| | <1,000 g), n (%) | 68 (41.5) | 81 (38.0) | 0.499 |
| | SGA (<10th), n (%) | 48 (29.3) | 51 (23.7) | 0.288 |
| | FGR/IUGR , n (%) | 6 (3.7) | 19 (8.9) | 2.6 (1.0–6.6), 0.049 |
| Male sex, n (%) | | 87 (53.0) | 119 (55.9) | 0.586 |
| Risk factors at birth | | | | |
| PPROM, n (%) | | 58 (35.4) | 49 (23.0) | 0.54 (0.34–0.86), 0.011 |
| PPROM >3 d, n (%) 74 | | 52 (31.7) | 22 (10.3) | 0.25 (0.14-0.43), <0.0001 |
| Delivery by cesarean section, n (%) | | 95 (57.9) | 109 (51.2) | 0.377 |
| Apgar 1, Median (Q1, Q3) | | 5 (2, 7) | 5 (2, 7) | 0.586 |
| Apgar 5, Median (Q1, Q3) | | 7 (6, 8) | 7 (6, 8) | 0.451 |
| Parameters related to respiratory failure | | | | |
| Surfactant treatment, n (%) | | 83 (50.6) | 98 (46.0) | 0.406 |
| Resuscitation, n (%) | | 133 (81.1) | 188 (86.4) | 0.164 |
| Mech. vent., n (%) | | 124 (75.6) | 134 (62.9) | 0.64 (0.43–0.95), 0.009 |
| Mech. vent. period [d], Median (Q1, Q3) | | 20 (6, 46) | 11 (1, 30) | 0.0001 |
| Oxygen supply period [d], Median (Q1, Q3) | | 28 (1, 58) | 13 (2, 59) | 0.020 |
| Blood transfusions, Mean (SD) | | 3.6 (3.4) | 3.6 (3.2) | 0.892 |
| Complications of prematurity, n (%) | | | | |
| NEC | | 27 (16.5) | 52 (24.4) | 0.062 |
| BPD | | 59 (36.0) | 90 (42.3) | 0.217 |
| IVH | | 92 (55.8) | 126 (59.2) | 0.551 |
| RDS | | 78 (47.6) | 173 (81.2) | 4.8 (3.0-7.6), <0.0001 |
| PVL | | 14 (8.5) | 14 (6.6) | 0.472 |
| ROP | | 102 (62.2) | 137 (64.3) | 0.671 |
| pROP | | 43 (26.2) | 77 (36.2) | 1.6 (1.0-2.5), 0.041 |
| PDA | | 39 (23.8) | 65 (30.5) | 0.148 |
| Death | | 3 (1.8) | 5 (2.3) | 0.730 |

Abbreviations and symbols: BPD – bronchopulmonary dysplasia; FGR – fetal growth restriction; IUGR - intrauterine growth retardation; IVH – intraventricular hemorrhage; NEC – necrotizing enterocolitis; PDA - patent ductus arteriosus; PPROM - preterm prelabor rupture of membranes; PVL - periventricular leukomalacia; RDS – respiratory distress syndrome; ROP – retinopathy of prematurity; SGA - small for gestational age.

**Supplemental Table S2.** Comparison of the impact of neonatal *MTHFR* rs1801133 genotypes on ELBW and ELGA in males and females. The table presents genotype (CT and TT vs CC) and allele-based comparisons (dominant and recessive models) with two-sided $\chi^2$ test p-values. Statistically significant values are highlighted in bold.

| Genotype and allele frequencies | ELBW | | | | ELGA | | | |
|---|---|---|---|---|---|---|---|---|
| | Female | | Male | | Female | | Male | |
| | no n=92 | Yes n=79 | no n=130 | Yes n=76 | no n=100 | Yes n=71 | no n=125 | Yes n=81 |
| *MTHFR* rs1801133C>T (Ala222Val) | | | | | | | | |
| *CC* | 50 (54.3) | 35 (44.3) | 75 (57.7) | 33 (43.4) | 50 (50.0) | 35 (49.3) | 72 (57.6) | 36 (44.4) |
| *CT* | 36 (39.1) | 33 (41.8) | 42 (32.3) | 35 (47.4) | 39 (39.0) | 30 (42.3) | 40 (32.0) | 38 (46.9) |
| *TT* | 7 (6.5) | 11 (13.9) | 13 (10.0) | 7 (9.2) | 11 (11.0) | 6 (8.5) | 13 (10.4) | 7(8.6) |
| MAF | 0.261 | 0.348 | 0.262 | 0.329 | 0.305 | 0.296 | 0.264 | 0.321 |
| Statistical analysis: OR (95%CI), p | | | | | | | | |
| Genotype | | | | | | | | |
| *CC* | 1.0 (reference) | | 1.0 (reference) | | 1.0 (reference) | | 1.0 (reference) | |
| *CT* | 1.31 (0.69–2.48); p = 0.409 | | **1.89 (1.03–3.48); p = 0.038** | | 1.10 (0.58–2.09); p = 0.774 | | **1.90 (1.05–3.45); p = 0.034** | |
| *TT* | 2.24 (0.79–6.36); p = 0.122 | | 1.22 (0.45–3.35); p = 0.694 | | 0.78 (0.26–2.30); p = 0.652 | | 1.08 (0.40–2.93); p = 0.885 | |
| Genetic model | | | | | | | | |
| Dominant (CT+TT vs CC) | 1.46 (0.80–2.67); p = 0.216 | | 1.74 (0.98–3.08); p = 0.059 | | 1.03 (0.56–1.89); p = 0.928 | | 1.70 (0.97–2.98); p = 0.065 | |
| Recessive (TT vs CT+CC) | 1.99 (0.73–5.40); p = 0.172 | | 0.93 (0.35–2.43); p = 0.877 | | 0.75 (0.26–2.12); p = 0.583 | | 0.81 (0.31–2.14); p = 0.677 | |

**Supplemental Table S3.** Meta-analysis results of association of *MTHFR* variant with LBW.

| Variant/Analysis | Genetic model | | Type of model | Heterogeneity | | Statistical analysis | | | | Publication bias; $p_{Eggers}$ |
|---|---|---|---|---|---|---|---|---|---|---|
| | | | | I² (%) | $p_H$ | OR | 95% CI | $p_{crude}$ | $p_{adj}$ | |
| *MTHFR* rs1801133T | Allele contrast | T vs. C | REM | 79 | 0.001 | 1.10 | 0.80; 1.45 | 0.607 | 1.000 | 0.897 |
| | Recessive | TT vs. TC+CC | REM | 60 | 0.039 | 1.14 | 0.84; 1.95 | 0.245 | 1.000 | 0.719 |
| | Dominant | TT+TC vs. CC | REM | 70 | 0.010 | 0.97 | 0.70; 1.42 | 0.996 | 1.000 | 0.779 |
| | Overdominant | TC vs. TT+CC | REM | 68 | 0.013 | 0.83 | 0.59; 1.13 | 0.219 | 1.000 | 0.719 |
| | Homozygotes 1 | TT vs. CC | REM | 59 | 0.046 | 1.18 | 0.77; 1.93 | 0.399 | 1.000 | 0.735 |
| | **Homozygotes 2** | **TT vs. TC** | **FEM** | **3** | **0.395** | **1.41** | **1.08; 1.80** | **0.0097** | **0.068** | **0.949** |
| | Heterozygotes | TC vs. CC | REM | 28 | 0.228 | 0.92 | 0.64; 1.30 | 0.856 | 1.000 | 0.152 |
| *MTHFR* rs1801133T Developed countries | Allele contrast | T vs. C | FEM | 0 | 0.617 | 0.85 | 0.71; 1.00 | 0.054 | 0.378 | n/a |
| | Recessive | TT vs. TC+CC | FEM | 0 | 0.886 | 0.88 | 0.61; 1.27 | 0.503 | 1.000 | n/a |
| | **Dominant** | **TT+TC vs. CC** | **FEM** | **0** | **0.434** | **0.79** | **0.63; 0.99** | **0.038** | **0.267** | **n/a** |
| | Overdominant | TC vs. TT+CC | FEM | 0 | 0.408 | 0.82 | 0.65; 1.03 | 0.086 | 0.599 | n/a |
| | Homozygotes 1 | TT vs. CC | FEM | 0 | 0.885 | 0.79 | 0.54; 1.16 | 0.226 | 1.000 | n/a |
| | Homozygotes 2 | TT vs. TC | FEM | 0 | 0.673 | 1.01 | 0.69; 1.50 | 0.947 | 0.068 | n/a |
| | Heterozygotes | TC vs. CC | FEM | 0 | 0.377 | 0.79 | 0.63; 0.99 | 0.049 | 0.346 | n/a |
| *MTHFR* rs1801133T Developing countries and this study | Allele contrast | T vs. C | FEM | 21 | 0.282 | 1.43 | 1.18; 1.73 | <0.0001 | 0.002 | 0.027 |
| | Recessive | **TT vs. TC+CC** | **FEM** | **0** | **0.558** | **1.85** | **1.34; 2.53** | **<0.0001** | **0.001** | **0.548** |
| | Dominant | TT+TC vs. CC | FEM | 50 | 0.138 | 1.38 | 1.02; 1.85 | 0.034 | 0.237 | 0.041 |
| | Overdominant | TC vs. TT+CC | REM | 83 | 0.003 | 0.80 | 0.40; 1.62 | 0.535 | 1.000 | 0.876 |
| | Homozygotes 1 | TT vs. CC | FEM | 0 | 0.763 | 1.87 | 1.24; 2.81 | 0.003 | 0.019 | 0.259 |
| | Homozygotes 2 | TT vs. TC | FEM | 36 | 0.208 | 1.78 | 1.27; 2.49 | 0.001 | 0.005 | 0.885 |
| | Heterozygotes | TC vs. CC | REM | 67 | 0.049 | 1.05 | 0.58; 1.90 | 0.868 | 1.000 | 0.183 |

Abbreviations: FEM - the fixed effects model; REM - the random effects model.
